# Timing and effect of a safe routes to school program on child pedestrian injury risk during school travel hours: Bayesian changepoint and difference-in-differences analysis

**DOI:** 10.1186/s40621-014-0017-0

**Published:** 2014-07-29

**Authors:** Charles DiMaggio, Qixuan Chen, Peter A Muennig, Guohua Li

**Affiliations:** 1 Columbia University College of Physicians and Surgeons Department of Anesthesiology, Mailman School of Public Health Department of Epidemiology; Center for Injury Epidemiology and Prevention, Columbia University Medical Center, 622 West 168 Street, New York, NY 10032 USA; 2 Mailman School of Public Health Department of Biostatistics, 722 West 168 Street, New York, NY 10032 USA; 3 Mailman School of Public Health Department of Health Policy and Management, 722 West 168 Street, New York, NY 10032 USA

**Keywords:** Pediatric, Pedestrian, Injury, School travel, Changepoint, Bayesian

## Abstract

**Background:**

In 2005, the US Congress allocated $612 million for a national Safe Routes to School (SRTS) program to encourage walking and bicycling to schools. We evaluated the effectiveness of a SRTS in controlling pedestrian injuries among school-age children.

**Methods:**

Bayesian changepoint analysis was applied to model the quarterly counts of pedestrian injuries among 5- to 19-year old children in New York City between 2001 and 2010 during school-travel hours in census tracts with and without SRTS. Overdispersed Poisson model was used to estimate difference-in-differences in injury risk between census tracts with and without SRTS following the changepoint.

**Results:**

In SRTS-intervention census tracts, a change point in the quarterly counts of injuries was identified in the second quarter of 2008, which was consistent with the timing of the implementation of SRTS interventions. In census tracts with SRTS interventions, the estimated quarterly rates of pedestrian injury per 10,000 population among school-age children during school-travel hours were 3.47 (95% Credible Interval [CrI] 2.67, 4.39) prior to the changepoint, and 0.74 (95% CrI 0.30, 1.50) after the changepoint. There was no change in the average number of quarterly injuries in non-SRTS census tracts. Overdispersed Poisson modeling revealed that SRTS implementation was associated with a 44% reduction (95% Confidence Interval [CI] 87% decrease to 130% increase) in school-age pedestrian injury risk during school-travel hours.

**Conclusions:**

Bayesian changepoint analysis of quarterly counts of school-age pedestrian injuries successfully identified the timing of SRTS intervention in New York City. Implementation of the SRTS program in New York City appears to be effective in reducing school-age pedestrian injuries during school-travel hours.

**Electronic supplementary material:**

The online version of this article (doi:10.1186/s40621-014-0017-0) contains supplementary material, which is available to authorized users.

## 1 Background

In 2005, to help address the health and societal consequences of the decline in walking and bicycling to school, the US Congress created the federal Safe Routes to School (SRTS) program as part of the federal Safe, Accountable, Flexible and Efficient Transportation Equity Act (SAFETEA). The program allocated $612 million dollars for fiscal years 2005 to 2009 for state departments of transportation to build sidewalks, bicycle lanes, safe crossings, improve signage and make other improvements to the built environment to allow children to more safely travel to school (Safe Routes to School National Partnership [2012]). As of 2010, departments of transportation in all 50 states had introduced safety improvements at 10,400 of the nation’s 98,706 elementary and secondary schools. Interventions consist primarily of sidewalk improvements (19%), traffic calming (14%), pedestrian/bicycle access (14%), and education (14%). The distribution of projects mirrored the population density of school-age children across the United States (National Center for Safe Routes to School [2012]).

New York State received $31 million dollars from the 2005 SAFETEA SRTS federal budget allocation of which $10,298,000 was allocated to New York City (National Center for Safe Routes to School [2013]). As part of this funding, the New York City Department of Transportation introduced safety improvements at 124 schools with the highest injury rates. The work included traffic calming measures like narrowing roads, new traffic and pedestrian signals, the addition of timed crossings that allow pedestrians to cross before cars, speed bumps, speed boards (radar-equipped digital signs that tell drivers how fast they are moving), high visibility crosswalks and new parking regulations. These changes reduce pedestrian injuries by slowing traffic, ceding rights to pedestrians, and providing disincentives for driving. As of 2009, the New York City Department of Transportation reported that “100% of the short-term safety improvements … are complete”, and that additional longer term capital improvements had advanced far enough for the department of transportation to propose expanding the program to an additional group of approximately 100 schools (New York City Department of Transportation [2012]).

A number of studies have demonstrated the impact of SRTS programs on children’s physical activity, such as walking and biking to school, but less is known about the effectiveness of the SRTS program in reducing pedestrian injury risk in school-age children (Cradock et al. [2012]; Chriqui et al. [2012]; Levin Martin et al. [2009]). In a previous analysis of an SRTS program in New York City, we demonstrated the association of SRTS interventions with decreased pediatric pedestrian injury risk (DiMaggio and Li [2013]). In this paper, we present a Bayesian changepoint approach to more precisely determine if and when the change in risk was associated with the timing of the program, and measure the extent to which the post-changepoint risk declined in SRTS compared to non-SRTS areas.

## 2 Methods

### 2.1 Data sources

Motor-vehicle crash data were obtained from the New York City Department of Transportation. The data were based on police investigations for all crashes in New York City involving “death, personal injury or property damage to any one person in excess of $1,000” for the years 2001 to 2010. The data were entered by an investigating law enforcement officer onto a form (MV104AN) and abstracted into a Microsoft Access database by personnel of the New York City Department of Transportation.

Both pedestrians and bicyclists were included in the analysis, though only 0.7% of crashes were coded as bicyclists. School-age children were defined as those 5 to 19 years old at the time of injury as listed on the crash report. The data were read into the R statistical analysis program (R Development Core Team [2011]), and evaluated for outliers, inconsistent values and missing entries.

Date and time variables were translated into Portable Operating System Interface (POSIX) time objects to extract variables for year, month, day and hour. A school-time indicator variable was created to identify crashes that occurred during days and hours when school-age children would be expected to be traveling to or from school, defined as 7 AM to 9 AM or 2 PM to 4 PM, Monday to Friday between September and June. A geographic variable was created using crash latitude and longitude coordinates, and were assigned to census tracts using the R maptools package (Lewin-Koh Roger [2012]).

The SRTS school location data were similarly obtained from the New York City Department of Transportation, and consisted of ArcGIS (ESRI, Redlands, California, USA) files for 124 New York City schools selected by the Department of Transportation for SRTS interventions. These schools, chosen from among the city's 1,471 schools, had the highest rates of pedestrian injury. The New York City Department of Transportation developed a ranking system to prioritize schools for SRTS interventions based on school-age pedestrian crash counts and injury severity within a 700-foot radius of geocoded school locations for the three-year period 1998–2000. A detailed description is available (The RBA Group Urbitran Associates, Inc [2013]). By 2009, 12 schools had completed short-term intervention measures such as new crosswalk markings, and replaced or improved signage and had completed capital construction projects; 18 schools had completed short-term interventions and had capital construction under way; and 94 schools had either started or planned to start interventions, but had not yet started capital construction.

The 12 schools that had completed both short-term measures and capital construction by 2009 and the 18 schools that had completed short-term interventions and had capital construction projects ongoing by 2009 were combined into a group of 30 SRTS intervention schools located within 30 census tracts. For comparison, the non-intervention census tracts were defined as those containing schools that were not included as one of the 124 SRTS schools. While the implemented interventions differed somewhat from one census tract to the other, our goal was to measure the net effect across interventions rather than the efficacy of any given measure.

Because there was insufficient information to accurately determine whether SRTS interventions had been completed, we excluded from the changepoint model 94 census tracts that contained schools that may or may not have started short-term interventions, and had not yet started capital construction projects at the time the study was conducted. These sites were used for sensitivity analysis of the overdispersed Poisson difference-in-differences model. Population data were based on age-stratified United States Census files from both the 2000 and 2010 decennial census at the census tract level (US Census Bureau [2010]). The population count at the census tract level was extrapolated over the intervening decade using linear interpolation. Counts of pedestrian crashes involving school-age children during school-travel hours were aggregated by year and quarter and stratified by intervention vs. non-intervention census tracts.

### 2.2 Bayesian changepoint model

In a Bayesian approach, we base our conclusions about the probability of a value for a parameter given our data, Pr(θ|y), on a combination of our prior expectation for the value of that parameter, expressed as the probability of observing the parameter Pr(θ), and the likelihood of observing the data we have collected, Pr(y|θ) given that parameter:

1 $$\mathrm{Pr}\left(\theta |y\right)\mathrm{\propto Pr}\left(y|\theta \right)*\mathrm{Pr}\left(\theta \right),$$

For a changepoint problem, we are interested in estimating the time point in which a change occurred in a time series, conditioned on the data we have observed (Albert [2008]). We assume that at some critical ‘changepoint’, t = τ, during our 10 years of observation, the rate of school-travel pediatric pedestrian crashes changed.

The data series in this study were *y*_t_ and O_t_*,* the quarterly count of school-age, school-hour pedestrian injuries and population offset at time period t. The data were assumed to be Poisson distributed and were modeled using Model (1) for census tracts with SRTS intervention and using Model (2) for census tracts without SRTS intervention:

2 $$y_{t}\sim \mathrm{Poi}\left(\mu_{t}\right),$$

3 $$\log\left(\mu_{t}\right)=\beta_{0}+\beta_{1}t+\beta_{2}\delta \left(t–\tau \right)+\beta_{3}\delta \left(t–\tau \right)\left(t–\tau \right)+\log\left(O_{t}\right),$$

4 $$\log\left({µ}_{t}\right)=\beta_{0}+\beta_{2}\delta \left(t–\tau \right)+\log\left(O_{t}\right),$$

5 t=1,…,40.

A binary 1/0 changepoint function, δ, is defined as 1 only if its argument is non-negative (t ≥ τ). Model (1) assumes that both the intercept and the slope of the Poisson model changed post τ, while Model (2) assumes no time effect (β_1_ = β_3_ = 0) except the difference in the mean injury rate pre- and post-change. Model (2) is a special case of Model (1) with β_1_ = β_3_ = 0 and was fitted in the non-SRTS group because convergence could not be achieved using Model (1) and a linear time effect was not observed in this group. Completing the model description, β_0_ is the pre-change intercept, β_1_ is the pre-change slope, β_0_ + β_2_ is the post-change intercept, and β_1_ + β_3_ is the post-change slope. Working on the log scale, prior to t = τ the log mean injury rate at time t is equal to β_0_ + β_1_t and β_0_, for census tracts with and without SRTS interventions, respectively. On or after the changepoint, the log mean is hypothesized to reset to a new level, with β_0_ + β_2_ + β_1_t + β_3_(t-τ) and β_0_ + β_2_, for census tracts with and without SRTS interventions, respectively.

A prior distribution for the parameters in the model is defined to reflect uncertainty about whether there actually was a change in injury counts during the study period by placing a uniform distribution on the interval of 40 quarters of observation in census tracts with and without SRTS interventions for the changepoint τ, and a normal distribution centered at 0 for the beta coefficients:

6 $$\tau \sim U\left(1,40\right)$$

7 $$\beta_{0},\beta_{1},\beta_{2},\beta_{3}\sim N\left(0,10^{6}\right).$$

The model was evaluated with Monte Carlo Markov chain simulations using the JAGS Gibb’s sampler program (Plummer [2010]) under the R2jags interface (Su and Masanau [2012]).

Convergence to a stable posterior sample of values was assessed by visually inspecting the traceplot of simulated values and with the R coda package to calculate the Brooks-Gellman-Rubin statistic, which compares within chain variation to across chain variation (Plummer et al. [2012]). Injury count and population data, JAGS model syntax, and quarterly posterior means and statistics for SRTS-intervention sites can be found in the Additional file 1.

### 2.3 Poisson model

To assess the impact of SRTS interventions on school-travel pediatric pedestrian injuries following the proposed changepoint, we fit an overdispersed Poisson model (Gelman and Hill [2007]):

8 $$\log\left(\mu_{t}\right)=\beta_{0}+\beta_{1}{\mathrm{Period}}_{t}+\beta_{2}\mathrm{SRTS}+\beta_{3}{\mathrm{Period}}_{t}*\mathrm{SRTS}+\log\left(O_{t}\right),$$

Where "Period" refers to the pre vs. post changepoint time period, and "SRTS" is an indicator variable for the presence of SRTS interventions in a census tract. A single changepoint, based on the SRTS interventions census tracts was used in the model. The coefficient for the interaction term for time period and intervention status is a measure of the difference in differences between census tracts with and without SRTS interventions from the pre-changepoint to the post-changepoint time period. The inclusion of the population offset allows the exponentiated coefficients to be interpreted as incidence rate ratios.

The primary model compared the experience in census tracts with schools that had completed SRTS interventions to census tracts in which there were no schools that had completed SRTS interventions. As a sensitivity analysis, we conducted an additional analysis in which census tracts with schools that had completed SRTS interventions were compared to census tracts in which there was a school that had been identified for SRTS interventions, but for which no or only short-term or temporary interventions, such as lighting and signage replacement, had been completed. The study protocol was approved as exempt by the Columbia University Medical Center institutional review board.

## 3 Results

The full data set consisted of 140,835 pedestrian crashes, which occurred between 2001 and 2010 in 1,929 New York City census tracts. There were 35,587 pedestrian injuries among school-age children (ages 5 to 19). The largest proportion of school-age pedestrian injuries occurred during the non-school months of July and August (11.6% and 9.6% respectively). A total of 4,021 (11.3%) school-age pedestrian injuries occurred during school-travel hours (7 AM to 9 AM or 2 PM to 4 PM).

**Figure 1:**
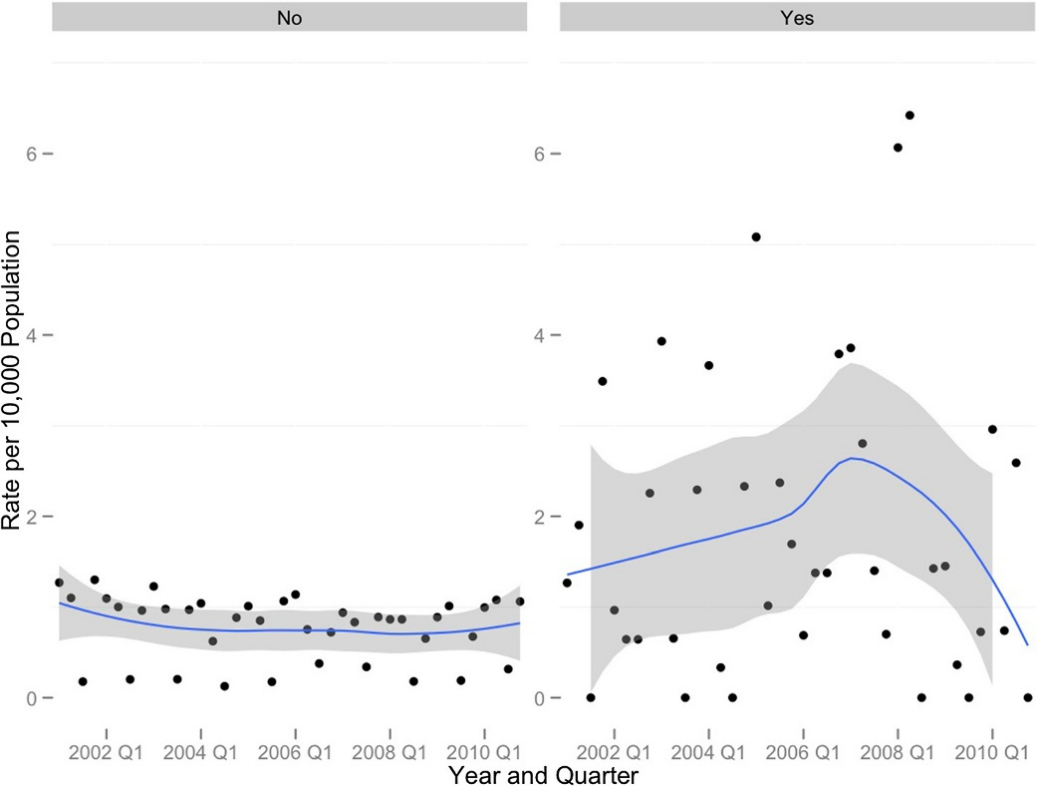
**Quarterly time series with loess trend line and 95% confidence band, school-aged pedestrian crashes per 10 000 population during school-travel (to and from) hours: Safe Routes to School intervention census tracts (yes) versus nonintervention census tracts (no), New York City, 2001–2010.**

In census tracts with SRTS interventions, the quarterly rate of school-age pedestrian injuries during school hours appeared to be increasing in the earlier years of the study period and then sharply declined at some point following 2008. There was no similar change in the quarterly time series for census tracts without SRTS interventions (Figure 1).

In the Bayesian changepoint analysis of the quarterly count data for the SRTS intervention group, the most likely changepoint occurred between the 30th and 31st quarter, which corresponds to the second or third quarter of 2008 (Table 1).

**Table 1:** **Results from the Bayesian changepoint analysis**

Variable	Posterior mean	95% CrI
SRTS Intervention		
Changepoint (δ), number of quarters from January 2001	30.5	(30.02, 30.98)
β0 (Pre-changepoint intercept)	1.25	(0.90, 1.58)
β1 (Pre-changepoint slope)	0.03	(0.02, 0.05)
β2 (Pre- and post intercept difference)	−1.68	(−2.59, −0.84)
β3 (Pre- and post slope difference)	0.04	(−0.1, 0.17)
β0 + β2 (Post-changepoint intercept)	−0.43	(−1.45, 0.52)
β1 + β3 (Post-changepoint slope)	0.07	(−0.06, 0.21)
Non-SRTS Intervention		
Changepoint (δ), number of quarters from January 2001	10.49	(10.02, 10.97)
β0 (Pre-changepoint intercept)	4.79	(4.74, 4.85)
β2 (Pre- and post intercept difference)	−0.29	(−0.36, −0.27)
β0 + β2 (Post-changepoint intercept)	4.50	(4.46, 4.53)

Posterior means and 95% Credible Intervals (CrI) on log scale. Safe Routes to School (SRTS) intervention census tracts and non-SRTS intervention census tracts, New York City, 2001–2010.

In the SRTS intervention areas, there were an estimated 3.66 (95% Credible Intervals [CrI] 2.58, 4.96) school-travel school-age injuries in the first quarter of 2001. This translates to a quarterly injury rate of 1.16 per 10,000 (95% CrI 0.84, 1.66) in the first quarter of 2001. The number of injuries increased until the changepoint of the second quarter of year 2008 (τ = 30). The injury count was estimated to be exp(0.03) *-1* = 3% (95% CrI 2%, 5%) higher for each quarter prior to the changepoint (Table 1). The average number of injuries in the quarter just before and after the changepoint was estimated to be 9.73 (95% CrI 7.49, 12.31) and 2.06 (95% CrI 0.84, 4.08), respectively. These counts translated to an estimated pre-changepoint quarterly rate of 3.47 per 10,000 (95% CrI 2.67, 4.39), and a post-changepoint quarterly rate of 0.74 per 10,000 (95% CrI 0.30, 1.5).

We contrasted these results to those for the non-SRTS intervention areas, which used a simplified model (2) to achieve convergence (Table 1). The most likely changepoint occurred between the 10^th^ and 11^th^ quarter, which corresponds to the 2^nd^ or 3^rd^ quarter of 2003. In the non-SRTS areas, the average number of pre-changepoint injuries was estimated to be 120.30 (95% CrI 114.43, 127.74). The average number of post-changepoint injuries was exp(4.50) = 90.02 (95% CrI 86.49, 92.76). These counts translate to pre and post changepoint quarterly injury rates of 0.96 (95% CrI, 0.92, 1.02) and 0.72 (95% CrI 0.69, 0.74) per 10,000 population.

**Figure 2:**
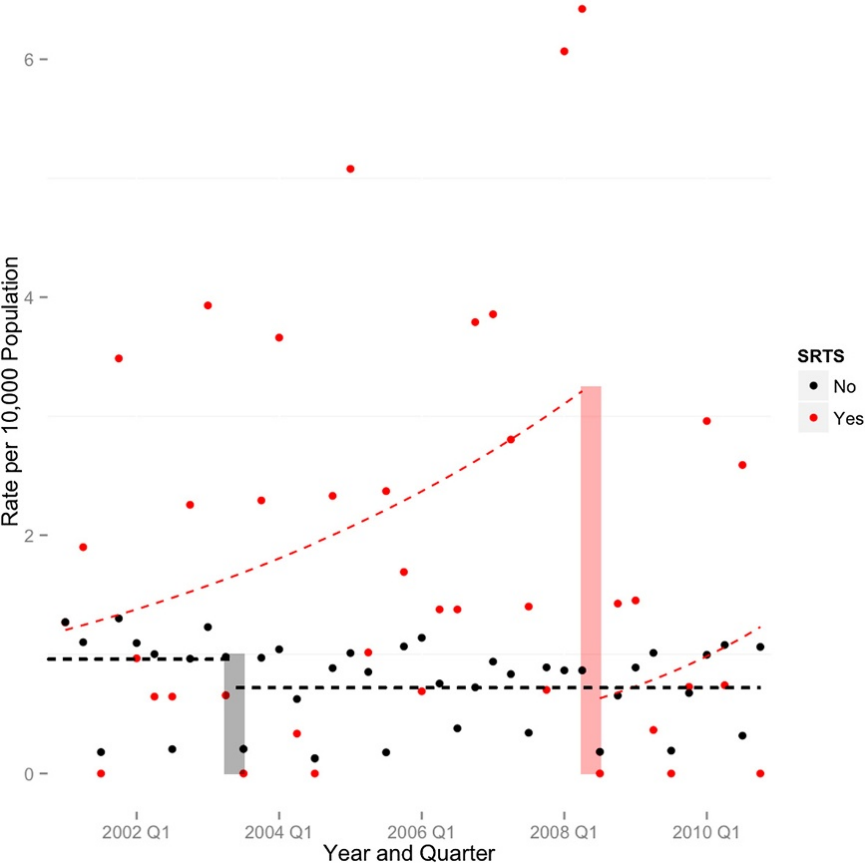
**Changepoint model fit, Non-Safe Routes to School (SRTS) intervention census tracts vs. SRTS intervention census tracts, school-age, school travel pedestrian injuries New York City, 2001–2010.**

The changepoint results for both the SRTS and non-SRTS areas are illustrated in Figure 2.

Table 2 presents the results of the overdispersed Poisson model. The β_3_ coefficient represents the difference between intervention and non-intervention census tracts in the incidence density ratio of injuries pre- and post-changepoint. In the model comparing census tracts with completed SRTS improvements to non-SRTS census tracts, there was a 1-exp(-0.58) = 44% overall risk reduction (95% Confidence Interval [CI] 87% decrease to 130% increase). In the model comparing census tracts with completed SRTS interventions to census tracts with incomplete SRTS interventions, there was an overall risk reduction of 32% (95% CI 74% decrease to 78% increase). The insignificance of the interaction term here might be due to small numbers of data points used in the regression model.

**Table 2:** **Regression coefficients and 95% confidence intervals from overdispersed Poisson models**

	Census Tracts with Completed SRTS Interventions Vs. Census Tracts with No SRTS Interventions	Census Tracts with Completed SRTS Interventions Vs. Census Tracts with Partially Completed SRTS Interventions
Variable		
β0	−9.43 (−9.55, −9.31)	−8.51 (−8.69, −8.33)
β1	0.95 ( 0.42, 1.48)	0.03 (−0.32, 0.38)
β2	−0.13 (−0.40, 0.14)	−0.33 (−0.76, 0.10)
β3	−0.58 (−2.01, 0.85)	−0.38 (−1.34, 0.58)

Coefficients and 95% Confidence Intervals (CI) on log scale. Completed Safe Routes to School(SRTS) interventions census tracts and non-SRTS intervention census tracts (model 1) and completed SRTS census tracts vs. not-completed SRTS intervention census tracts (model 2), New York City, 2001–2010.

## 4 Discussion

Though intended primarily to encourage physical activity among school children, the national SRTS program represents perhaps the largest single United States federal government expenditure for pediatric pedestrian safety in US history. In this study, SRTS interventions in New York City were associated with a decline in pedestrian injuries among school-age children traveling to or from school. The changepoint occurred at the time SRTS interventions were implemented, and followed years of increasing numbers of injuries.

Our primary intention in this paper was to precisely estimate the point at which declines in school-age, school-travel pedestrian injury in SRTS areas are likely to have occurred, and to estimate the change in risk based on this more precise estimate. A previous analysis of these data similarly demonstrated the association of SRTS interventions with decreased pediatric pedestrian injury risk, but one could reasonably interpret the time series as indicating changes prior to the implementation of interventions (DiMaggio and Li [2013]). The current analysis indicates the change was very likely to have occurred at or about the time SRTS interventions were implemented, and was associated with meaningful, though in this analysis not statistically significant, decreases in risk. These results argue for the effectiveness of SRTS interventions in controlling school-travel related pediatric pedestrian injury.

It should perhaps not come as a surprise that SRTS interventions are effective in preventing school-travel pedestrian injuries. Engineering approaches to injury control are often the most effective (Haddon [1980]), and SRTS legislation requires that 70 to 90% of SRTS funds be used for infrastructure projects like sidewalk construction, compared to 10 to 30% for education projects (Levin Martin et al. [2009]). Fully 63% of all SRTS projects nationwide involve some change to the built environment. Approximately 39% of urban land areas are within a half mile of a school, leading some investigators to conclude that SRTS programs have the potential to benefit 65.5 million people in the United States (Watson and Dannenberg [2008]).

Evaluations of change following policy interventions can be challenging. Sequential observations are not independent. Secular trends and seasonal variations make it difficult to draw conclusions based on simple pre-post comparisons. Bayesian methods have been proposed as effective approaches to changepoint problems (Carlin et al. [1992]), which address the identification of the point at which a change occurs in a time series. While not common in injury research, Bayesian changepoint methods have been applied to such public health questions as declines in violent assault following closings of alcohol outlets (Yu et al. [2008]), evaluating the clinical significance of serial CD4 counts (Ghosh and Vaida [2007]), and biomarker assays (Bellera et al. [2008]), and predicting cancer recurrence (Pauler and Finkelstein [2002]).

Bayesian changepoint methods hold several advantages for injury researchers. The approach lends itself to the setting where data are scarce, expectations are uncertain, and the underlying data series may be difficult to model using either deterministic or stochastic time series approaches. The Bayesian approach ‘treats the timing of change as uncertain” and allows the analyst to “discover” the changepoint from the data (Western and Kleykamp [2004]). Changepoint analysis also lends itself naturally to hierarchical modeling, since the parameters for prior distributions may be assigned their own prior distributions. This can allow us to incorporate additional sources of uncertainty, such as changepoints varying across geographic regions. This method also has the advantage of returning direct probability statements that may be more easily interpretable by policy makers (Kim and Nelson [1999]).

One of the drawbacks to a changepoint analysis is that the approach is inherently limited to a single series of observations. Our motivation for this changepoint analysis was to establish the temporal relationship between the SRTS interventions in the target population, and to contrast that to the lack of such a temporal relationship in a non-target population. To accomplish this, we stratified our analyses by target group, and based our comparisons on two separate changepoint models. While, in general, a single model of all the data is preferable and less prone to bias, changepoint modeling does not lend itself easily or intuitively to that approach and this is an area of research (Seidou et al. [2007]; Cai et al. [2012]), and we believe a stratified approach is valid for the temporal conclusions we draw from the analysis.

### 4.1 Limitations

The study is subject to a number of important limitations. The demonstrated declines cannot be separated from underlying secular trends, and indeed declines in overall pedestrian injury rates predate the SRTS program. New York City, through the efforts of the City Department of Transportation and the Department of Health and Mental Hygiene, has made important strides in making the city safer for all road users. This renders it difficult to tease out the effects of any single program or intervention.

The City Department of Transportation has taken a proactive approach to making the city walkable and safe. Total traffic fatalities decreased 35% between 2001 and 2009, making 2009 the safest year on record in terms of traffic crashes. The department issued a landmark Pedestrian Safety Study and Action Plan and continues efforts to install 1,500 pedestrian signals, re-engineer 60 miles of streets and 20 intersections, evaluate 20 mile-per-hour pedestrian safety zones, and pilot a program to improve left-turn visibility in Manhattan (Viola et al. [2010]). Also, the target groups and areas started at higher injury rates than non-intervention groups, and there may have been some regression to the mean in the time series. Despite these caveats, there is evidence of a temporal change only in those areas in which there were completed interventions, occurring only when the interventions were completed, and associated with a decline in risk only in those areas, and only after the SRTS interventions were put in place.

To ensure the consistency and reliability of the population data across the 10 years of study, and across the analytic approaches that took geography into account, we restricted the analyses to those census tracts that were present in both census years. Because injury rates have been dropping over time, this resulted in slightly inflated absolute injury rates. However, it allowed for more valid comparisons across space and time, and between intervention and non-intervention sites.

Census tracts are most often not ideal geographies at which to measure traffic injury control interventions, and many such analyses use data based on intersections or geographic buffers based on an intersection. If exposures were based on traffic volume, or if the interventions were limited to single sites, then an intersection-based approach might make more sense. That was not the case with these interventions. The New York City Department of Transportation describes the extent of SRTS interventions to be approximately 800 feet surrounding a school, and it is this somewhat diffuse extent that may contribute to overall traffic calming in the area around a school. We note that the average area of a census tract in New York City is 90 acres or 3,920,400 square feet. An 800-foot circular geographic buffer would cover about  2,010,619 square feet. We note as well that our exposures are population-based, and that census tracts, which are defined to have stable underlying population estimates of between 3,000 and 4,000 may be more appropriate in this setting.

There were also important and significant changes in the demographics and population of New York City from 2000 to 2010. While there was an 2.08% increase (from 8,008,278 to 8,175,133 ) in the overall city population, there was at the same time a 9.2% decrease (1,612,572 to 1,477,146) in the number school-age children as we defined them for this study (US Census Bureau [2010]). While we do not have any data on the number of children actually walking to school, SRTS programs have been consistently demonstrated to increase the number of children walking to school, so the population exposure may likely have increased following the interventions.

There were also changes in the geographic make up of New York City during the study period. There were 2,217 census tracts in 2000, and 2,168 tracts in 2010, for an overall decrease of 2.2%. There were 288 census tracts in the 2000 data set that were not in the 2010 data set. There were conversely 239 census tracts in the 2010 data set that were not in the 2000 data set.

Finally, given that the interventions were not consistent across all intervention sites, our effect sizes measure net effects rather than the effect of any given intervention. It is likely that the Department of Transportation picked the most needed interventions for a given census tract. Therefore, we could not expect the findings to be generalizable to planners with superior or inferior data.

## 5 Conclusions

Manipulating the physical environment is an effective, though often difficult and expensive, approach to pedestrian injury control (DiMaggio and Li [2012]). Separating play areas from roadways, improved visibility at intersections, conspicuous stop signs, enhanced pavement markings and improved lighting prevent pedestrian injury. All of these interventions are represented by the New York City SRTS program, which in these data has been shown to be associated with a decline in school-travel related pediatric pedestrian injury risk in areas with completed SRTS interventions timed to the completion of those interventions.

## Electronic supplementary material

Additional file 1: **Appendices.** (DOCX 118 KB)

Below are the links to the authors’ original submitted files for images.

Authors’ original file for figure 1

Authors’ original file for figure 2
