# Supplementary material for: Timing and effect of a safe routes to school program on child pedestrian injury risk during school travel hours: Bayesian changepoint and difference-in-differences analysis
Source: Inj Epidemiol. 2014 Jul 29;1(1):17. doi: 10.1186/s40621-014-0017-0 (PMC5005758; doi:10.1186/s40621-014-0017-0)
Supplement: Supplementary file 1 — Additional file 1: Appendices. (DOCX 118 KB) [file 40621_2014_17_MOESM1_ESM.docx]

**Appendices**

Appendix 1: Quartely injury counts, population estimates and injury rates per 10,000. School-age pedestrian injuries during school travel hours, New York City , 2001-2010. Changepoints indicated by greyed cells.

|  | SRTS Interventions | | | No SRTS Interventions | | |
| --- | --- | --- | --- | --- | --- | --- |
| Quarter | Injury Count | Population | Rate | Injury Count | Population | Rate |
| 2001 Q1 | 4 | 31536 | 1.27 | 165 | 1297459 | 1.27 |
| 2001 Q2 | 6 | 31536 | 1.90 | 143 | 1297459 | 1.10 |
| 2001 Q3 | 0 | 31536 | 0.00 | 23 | 1297459 | 0.18 |
| 2001 Q4 | 11 | 31536 | 3.49 | 169 | 1297459 | 1.30 |
| 2002 Q1 | 3 | 31031 | 0.97 | 141 | 1287126 | 1.10 |
| 2002 Q2 | 2 | 31031 | 0.64 | 129 | 1287126 | 1.00 |
| 2002 Q3 | 2 | 31031 | 0.64 | 26 | 1287126 | 0.20 |
| 2002 Q4 | 7 | 31031 | 2.26 | 124 | 1287126 | 0.96 |
| 2003 Q1 | 12 | 30528 | 3.93 | 157 | 1276777 | 1.23 |
| 2003 Q2 | 2 | 30528 | 0.66 | 125 | 1276777 | 0.98 |
| 2003 Q3 | 0 | 30528 | 0.00 | 26 | 1276777 | 0.20 |
| 2003 Q4 | 7 | 30528 | 2.29 | 124 | 1276777 | 0.97 |
| 2004 Q1 | 11 | 30029 | 3.66 | 132 | 1266430 | 1.04 |
| 2004 Q2 | 1 | 30029 | 0.33 | 79 | 1266430 | 0.62 |
| 2004 Q3 | 0 | 30029 | 0.00 | 16 | 1266430 | 0.13 |
| 2004 Q4 | 7 | 30029 | 2.33 | 112 | 1266430 | 0.88 |
| 2005 Q1 | 15 | 29524 | 5.08 | 127 | 1256082 | 1.01 |
| 2005 Q2 | 3 | 29524 | 1.02 | 107 | 1256082 | 0.85 |
| 2005 Q3 | 7 | 29524 | 2.37 | 22 | 1256082 | 0.18 |
| 2005 Q4 | 5 | 29524 | 1.69 | 134 | 1256082 | 1.07 |
| 2006 Q1 | 2 | 29022 | 0.69 | 142 | 1245759 | 1.14 |
| 2006 Q2 | 4 | 29022 | 1.38 | 94 | 1245759 | 0.75 |
| 2006 Q3 | 4 | 29022 | 1.38 | 47 | 1245759 | 0.38 |
| 2006 Q4 | 11 | 29022 | 3.79 | 90 | 1245759 | 0.72 |
| 2007 Q1 | 11 | 28521 | 3.86 | 116 | 1235413 | 0.94 |
| 2007 Q2 | 8 | 28521 | 2.80 | 103 | 1235413 | 0.83 |
| 2007 Q3 | 4 | 28521 | 1.40 | 42 | 1235413 | 0.34 |
| 2007 Q4 | 2 | 28521 | 0.70 | 110 | 1235413 | 0.89 |
| 2008 Q1 | 17 | 28020 | 6.07 | 106 | 1225063 | 0.87 |
| 2008 Q2 | 18 | 28020 | 6.42 | 106 | 1225063 | 0.87 |
| 2008 Q3 | 0 | 28020 | 0.00 | 22 | 1225063 | 0.18 |
| 2008 Q4 | 4 | 28020 | 1.43 | 80 | 1225063 | 0.65 |
| 2009 Q1 | 4 | 27517 | 1.45 | 108 | 1214729 | 0.89 |
| 2009 Q2 | 1 | 27517 | 0.36 | 123 | 1214729 | 1.01 |
| 2009 Q3 | 0 | 27517 | 0.00 | 23 | 1214729 | 0.19 |
| 2009 Q4 | 2 | 27517 | 0.73 | 82 | 1214729 | 0.68 |
| 2010 Q1 | 8 | 27016 | 2.96 | 120 | 1204384 | 1.00 |
| 2010 Q2 | 2 | 27016 | 0.74 | 130 | 1204384 | 1.08 |
| 2010 Q3 | 7 | 27016 | 2.59 | 38 | 1204384 | 0.32 |
| 2010 Q4 | 0 | 27016 | 0.00 | 128 | 1204384 | 1.06 |

Appendix 2: Changepoint model code

model

{

for (i in 1:N)

{

Count[i] ~ dpois(mu[i])

log(mu[i]) <- b0 + b1*i + b2*step(i-tau) + b3*step(i-tau)*(i-tau)

}

b0 ~ dnorm(0, 1.0E-6)

b1 ~ dnorm(0, 1.0E-6)

b2 ~ dnorm(0, 1.0E-6)

b3 ~ dnorm(0, 1.0E-6)

tau ~ dunif(1,N)

intercept2 <- b0 + b2

slope2 <- b1 + b3

}

Appendix 3. The posterior mean, standard deviation, and some selected quantiles of the mean injury count at each quarter of years 2001-2010, calculated based on the Bayesian changepoint model (1) for census tracts with SRTS intervention.

| Quarter | mean | sd | 2.50% | 25.00% | median | 75.00% | 97.50% |
| --- | --- | --- | --- | --- | --- | --- | --- |
| 1 | 3.66 | 0.60 | 2.58 | 3.24 | 3.63 | 4.04 | 4.96 |
| 2 | 3.78 | 0.59 | 2.71 | 3.37 | 3.75 | 4.16 | 5.05 |
| 3 | 3.91 | 0.58 | 2.84 | 3.50 | 3.88 | 4.28 | 5.15 |
| 4 | 4.04 | 0.57 | 2.99 | 3.64 | 4.01 | 4.41 | 5.25 |
| 5 | 4.18 | 0.56 | 3.14 | 3.79 | 4.15 | 4.53 | 5.35 |
| 6 | 4.31 | 0.55 | 3.30 | 3.94 | 4.30 | 4.67 | 5.46 |
| 7 | 4.46 | 0.54 | 3.46 | 4.09 | 4.44 | 4.80 | 5.56 |
| 8 | 4.61 | 0.52 | 3.63 | 4.25 | 4.59 | 4.95 | 5.68 |
| 9 | 4.76 | 0.51 | 3.81 | 4.42 | 4.75 | 5.09 | 5.81 |
| 10 | 4.92 | 0.50 | 3.99 | 4.59 | 4.91 | 5.25 | 5.94 |
| 11 | 5.09 | 0.48 | 4.17 | 4.76 | 5.07 | 5.41 | 6.08 |
| 12 | 5.26 | 0.47 | 4.36 | 4.94 | 5.25 | 5.57 | 6.22 |
| 13 | 5.44 | 0.46 | 4.56 | 5.12 | 5.43 | 5.75 | 6.38 |
| 14 | 5.63 | 0.46 | 4.75 | 5.31 | 5.62 | 5.93 | 6.55 |
| 15 | 5.82 | 0.45 | 4.95 | 5.51 | 5.81 | 6.12 | 6.74 |
| 16 | 6.02 | 0.45 | 5.15 | 5.71 | 6.01 | 6.32 | 6.94 |
| 17 | 6.23 | 0.46 | 5.34 | 5.91 | 6.22 | 6.53 | 7.15 |
| 18 | 6.44 | 0.47 | 5.53 | 6.11 | 6.43 | 6.75 | 7.40 |
| 19 | 6.66 | 0.50 | 5.71 | 6.32 | 6.65 | 6.99 | 7.66 |
| 20 | 6.89 | 0.52 | 5.88 | 6.53 | 6.88 | 7.24 | 7.95 |
| 21 | 7.13 | 0.56 | 6.06 | 6.75 | 7.12 | 7.51 | 8.26 |
| 22 | 7.38 | 0.60 | 6.23 | 6.96 | 7.37 | 7.77 | 8.60 |
| 23 | 7.63 | 0.66 | 6.39 | 7.18 | 7.62 | 8.07 | 8.97 |
| 24 | 7.90 | 0.72 | 6.55 | 7.40 | 7.89 | 8.37 | 9.36 |
| 25 | 8.18 | 0.79 | 6.70 | 7.64 | 8.16 | 8.69 | 9.77 |
| 26 | 8.46 | 0.86 | 6.86 | 7.87 | 8.44 | 9.03 | 10.23 |
| 27 | 8.76 | 0.95 | 7.01 | 8.10 | 8.73 | 9.38 | 10.70 |
| 28 | 9.07 | 1.04 | 7.16 | 8.34 | 9.03 | 9.75 | 11.20 |
| 29 | 9.40 | 1.14 | 7.35 | 8.60 | 9.36 | 10.14 | 11.78 |
| 30 | 9.73 | 1.24 | 7.49 | 8.85 | 9.69 | 10.54 | 12.31 |
| 31 | 2.06 | 0.91 | 0.84 | 1.46 | 1.92 | 2.47 | 4.08 |
| 32 | 2.16 | 0.78 | 1.01 | 1.63 | 2.07 | 2.56 | 3.90 |
| 33 | 2.28 | 0.67 | 1.21 | 1.82 | 2.22 | 2.66 | 3.77 |
| 34 | 2.43 | 0.59 | 1.43 | 2.02 | 2.39 | 2.78 | 3.71 |
| 35 | 2.60 | 0.54 | 1.66 | 2.21 | 2.57 | 2.93 | 3.74 |
| 36 | 2.79 | 0.53 | 1.84 | 2.41 | 2.76 | 3.13 | 3.93 |
| 37 | 3.01 | 0.60 | 1.95 | 2.59 | 2.97 | 3.39 | 4.31 |
| 38 | 3.26 | 0.74 | 1.97 | 2.73 | 3.21 | 3.72 | 4.90 |
| 39 | 3.55 | 0.96 | 1.92 | 2.87 | 3.46 | 4.14 | 5.64 |
| 40 | 3.88 | 1.25 | 1.84 | 2.99 | 3.73 | 4.63 | 6.72 |
