# Supplementary figures and images for: Timing and effect of a safe routes to school program on child pedestrian injury risk during school travel hours: Bayesian changepoint and difference-in-differences analysis
Source: Inj Epidemiol. 2014 Jul 29;1(1):17. doi: 10.1186/s40621-014-0017-0 (PMC5005758; doi:10.1186/s40621-014-0017-0)

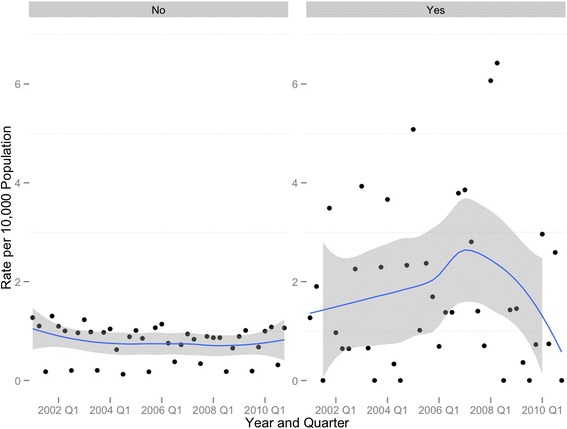

Supplement: Supplementary file 2 — Authors’ original file for figure 1 [file 40621_2014_17_MOESM2_ESM.gif]

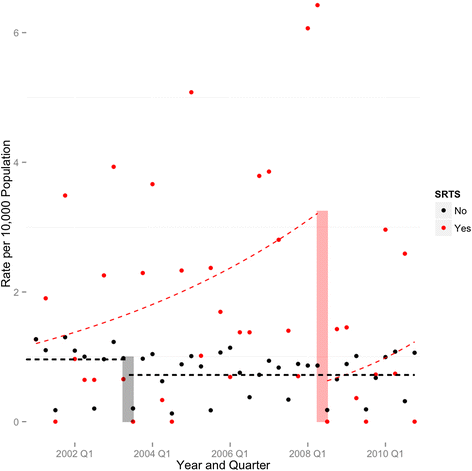

Supplement: Supplementary file 3 — Authors’ original file for figure 2 [file 40621_2014_17_MOESM3_ESM.gif]
